# Unlawful Practice of Medicine

**Published:** 1904-06

**Authors:** 


### Unlawful Practice of Medicine.

At a meeting of the New York Society of Medical Jurisprudence, held October 12, 1903, Justice Julius M. Mayer read a paper on "Criminal Procedure against the Unlawful practice of Medicine."

He reviewed the subject from the standpoint of a justice of the Court of Special Sessions, where cases of alleged unlawful practice are tried, and among his conclusions were: That a statute should be enacted defining more clearly what is the "practice of medicine," that there should be no minimum punishment provided in the law, and that some method should be devised to prevent the newspapers from publishing the advertisements of palmists and clairvoyants, whose real occupation is the practice of medicine illegally. In speaking on the latter point he said: "The worst agency in New York today that helps the man who sells either real or pretended abortion medicine is the newspapers, for they make it possible to ensnare the unwary, the superstitious and the fearful. I suggest that in the new school of journalism at Columbia University there be a chair of advertising, and let it be taught to the young men of the newspaper profession that the first duty of a great newspaper is to censor its medical advertising. Many persons who advertise as palmists and clairvoyants advertise simply to cover the giving of medicines of the kind I have mentioned."—*Medical Review of Reviews.*

